# The relationship between tissue oxygenation and redox status using magnetic resonance imaging

**DOI:** 10.3892/ijo.2012.1638

**Published:** 2012-09-24

**Authors:** FUMINORI HYODO, RYAN M. DAVIS, EMI HYODO, SHINGO MATSUMOTO, MURALI C. KRISHNA, JAMES B. MITCHELL

**Affiliations:** Radiation Biology Branch, Center for Cancer Research, National Cancer Institute, NIH, Bethesda, MD, USA

**Keywords:** redox status, hypoxia, electron paramagnetic resonance imaging, magnetic resonance imaging, nitroxide, non-invasive imaging, Tempol

## Abstract

The recent development of a bi-modality magnetic resonance imaging/electron paramagnetic resonance imaging (MRI/EPRI) platform has enabled longitudinal monitoring of both tumor oxygenation and redox status in murine cancer models. The current study used this imaging platform to test the hypothesis that a more reducing tumor microenvironment accompanies the development of tumor hypoxia. To test this, the redox status of the tumor was measured using Tempol as a redox-sensitive MRI contrast agent, and tumor hypoxia was measured with Oxo63, which is an oxygen-sensitive EPRI spin probe. Images were acquired every 1–2 days in mice bearing SCCVII tumors. The median pO_2_ decreased from 14 mmHg at 7 days after tumor implantation to 7 mmHg at 15 days after implantation. Additionally, the hypoxic fraction, defined as the percentage of the tumor that exhibited a pO_2_<10 mmHg, increased with tumor size (from 10% at 500 mm^3^ to 60% at 3,500 mm^3^). The rate of Tempol reduction increased as a function of tumor volume (0.4 min^−1^ at 500 mm^3^ to 1.7 min^−1^ at 3,500 mm^3^), suggesting that the tumor microenvironment became more reduced as the tumor grew. The results show that rapid Tempol reduction correlates with decreased tumor oxygenation, and that the Tempol decay rate constant may be a surrogate marker for tumor hypoxia.

## Introduction

Tumor hypoxia is prognostic for poor response to cancer therapy. The prognostic value of tumor hypoxia has been demonstrated in radiotherapy studies of human head and neck (1,2) and uterine cervix (3) tumors, which show that patients with less hypoxic tumors had a better chance of overall or disease-free survival than did patients with more hypoxic tumors. Furthermore, some chemotherapeutic agents such as bleomycin and doxorubicin exhibit a cytotoxicity that is strongly oxygen dependent, suggesting that hypoxia may be a prognostic factor for chemotherapeutic response as well (4–6). Therefore, development of methods allowing measurement of hypoxia in human patients may allow physicians to better manage tumors that exhibit considerable hypoxia.

Ilangovan *et al* demonstrated in tumor-bearing mice that carbogen breathing increased the oxygenation in the tumor, and that this increased oxygenation was related to a decreased rate of nitroxide reduction (7). Magnetic resonance imaging (MRI) can accurately measure nitroxide reduction rates (8,9), suggesting that nitroxide contrast agents could serve as an MRI-based assessment of tumor oxygenation. Although the study by Ilangovan showed that nitroxides were sensitive to oxygenation changes during carbogen breathing, another use of nitroxides would be to detect hypoxia in tumors (7). In this case, it is expected that hypoxia will have the opposite effect of carbogen on the rate of nitroxide reduction. Namely, it is expected that greater hypoxia will be associated with a greater rate of nitroxide reduction.

The purpose of this study was to test if there is a relationship between the reduction rate of Tempol as measured with MRI and the hypoxic fraction of a tumor. The hypoxic fraction of the tumor was measured using electron paramagnetic resonance (EPR) imaging and the triarylmethyl (TAM) spin probe Oxo63 and the reduction rate of Tempol was measured with a 7T small animal MRI scanner.

## Materials and methods

### Chemicals

The triarylmethyl (TAM) radical Oxo63 was obtained from GE healthcare. Tempol (4-hydroxy-2,2,6,6,-tetramethyl-1-piperidynyloxyl) was purchased from Sigma-Aldrich (St. Louis, MO, USA).

### Animals

C3HHenCrMTV- mice were obtained from the Frederick Cancer Research Center, Animal Production (Frederick, MD, USA). Mice were housed in a climate controlled circadian rhythm adjusted room and were allowed access to food and water *ad libitum*. The body weight of the mice at the time of imaging was 22–30 g. SCCVII (murine squamous cell carcinoma) cells (2–3×10^5^) were injected 7–15 days before imaging. Experiments were carried out in compliance with the Guide for the Care and Use of Laboratory Animal Resources (National Research Council, 1996) and approved by the National Cancer Institute Animal Care and Use Committee.

### Animal experiment protocol

Starting 7 days after injection of SCCVII cells, mice were imaged every 1–2 days. For anesthesia during imaging, a mixture of isofluorane (4% to induce, 1–2% to maintain) and medical air (750 ml/min) was blown into a nose-cone fitted to the animal’s head. For EPR measurements, the mouse was placed on a platform with its legs hanging downward into a vertical coil. For MRI measurements, the mouse was placed on a cradle, which was then placed inside the horizontal bore of the magnet. For both EPR and MRI experiments, a pressure transducer (SA Instruments Inc.) was used to monitor the breathing rate, and a rectal thermocouple was used to monitor the core body temperature. Warm air was blown over the mouse to maintain its temperature. The breathing rate was kept at 60±10 breaths per min and the body temperature was maintained at 37±1°C. To enable injection of contrast agents during imaging, a catheter was made by inserting a 30.5-gauge needle tip into polyethylene (PE-10) tubing. Using this catheter, the tail vein of the mouse was cannulated. Oxo63 (10 *μ*l/g body weight of 75 mM solution) or Tempol (5 *μ*l/g body weight of 150 mM solution) was manually injected through the catheter.

### Magnetic resonance imaging

Magnetic Resonance Imaging was performed using a 4.7 Tesla small animal scanner (Bruker Bio-Spin MRI GmbH). After a survey scan, T2-weighted images were obtained using multi-slice multi-echo (MSME) sequence with a 10-echo train and an echo time of 15 min. SPGR (also referred as gradient echo fast imaging, GEFI) (TR=75 ms, TE=3 ms, FA=45°, NEX=2) was employed to observe T1 effect. The scan time for 6 slices with the SPGR sequence was 20 sec. Other common image parameters are as follows; image resolution was 256 x 256, FOV was 3.2 x 3.2 cm, slice thickness was 2.0 mm. Number of slices was 6.

### EPR oximetry

A pulsed (time domain) EPR imaging scanner was used to measure tissue pO_2_. Details of this method are described elsewhere (10). Briefly, Oxo63 is injected into the mouse via tail vein catheter. Imaging was initiated once the EPR signal intensity reached a steady state value, indicating that the Oxo63 radical had reached a steady state concentration in the tissue. pO_2_ mapping relies on the linear relationship between the pO_2_ of the tissue and linewidth of the TAM radical (Oxo63.) To quantify the linewidth in a voxel, several images with increasing readout delay are obtained. As the readout delay increases, the signal decreases in a manner that depends on the linewidth of the voxel. From the images with successively increasing readout delay, the linewidth of oxo63 is mapped over the tissue region. Finally, using the linewidth map, the pO_2_ is calculated from a calibration curve calculated *in vitro*. EPR imaging was performed in a 300 MHz single point-imaging scanner. The imaging parameters were as follows: excitation pulse, 80 ns, 80 W; TR, 5.5 *μ*s; flip angle, 70°C; field gradient, 0.8 Gauss/cm, 1.0 Gauss/cm, 1.2 Gauss/cm; no. of gradient steps, 21 x 21, number of averages, 100,000.

### Statistical analyses

The statistical differences were estimated with TTEST function in the Microsoft Excel XP. The suitable ‘type’ for the test was selected according to the correspondence and variance of the data. Significances were estimated when p-value was less than 0.05.

## Results

### The growth of the SCCVII tumor as a function of time after implantation (Fig. 1A)

**Figure 1:**
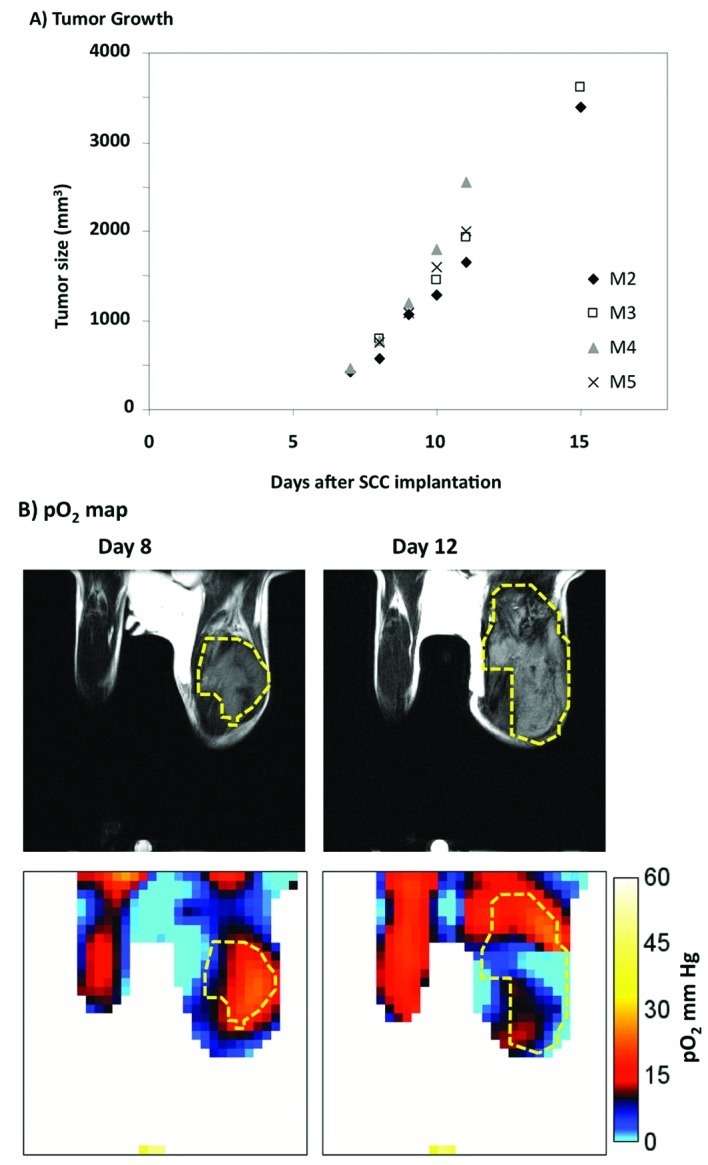
(A) SCCVII tumor size as a function of time after implantation. (B) T2-weighted anatomic image of a representative SCCVII tumor-bearing mouse at day 8 and day 12 (upper panels) and corresponding pO_2_ maps (bottom panels) acquired by EPRI. The tumor location is delineated by dashed yellow line on each of the images.

**Figure 2:**
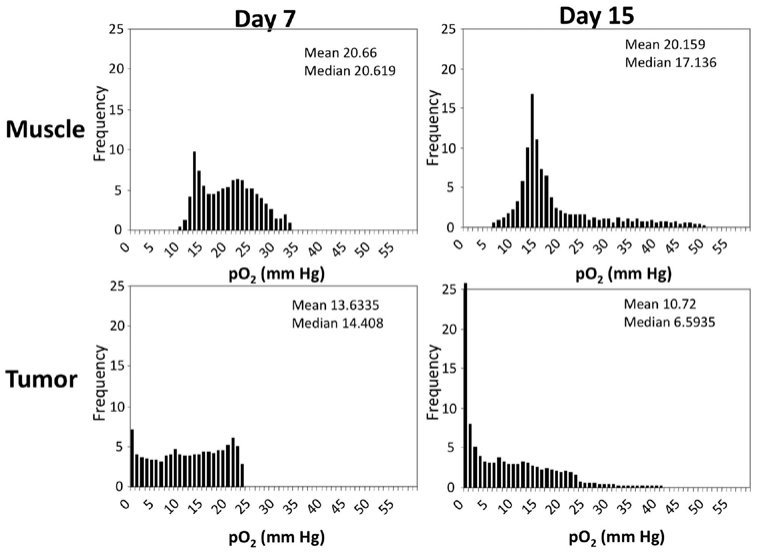
Comparison of muscle and tumor pO_2_ histograms at day 7 and day 15 from a representative SCCVII tumor-bearing mouse. Hypoxia increased substantially from day 7 to day 15 in the tumor.

**Figure 3:**
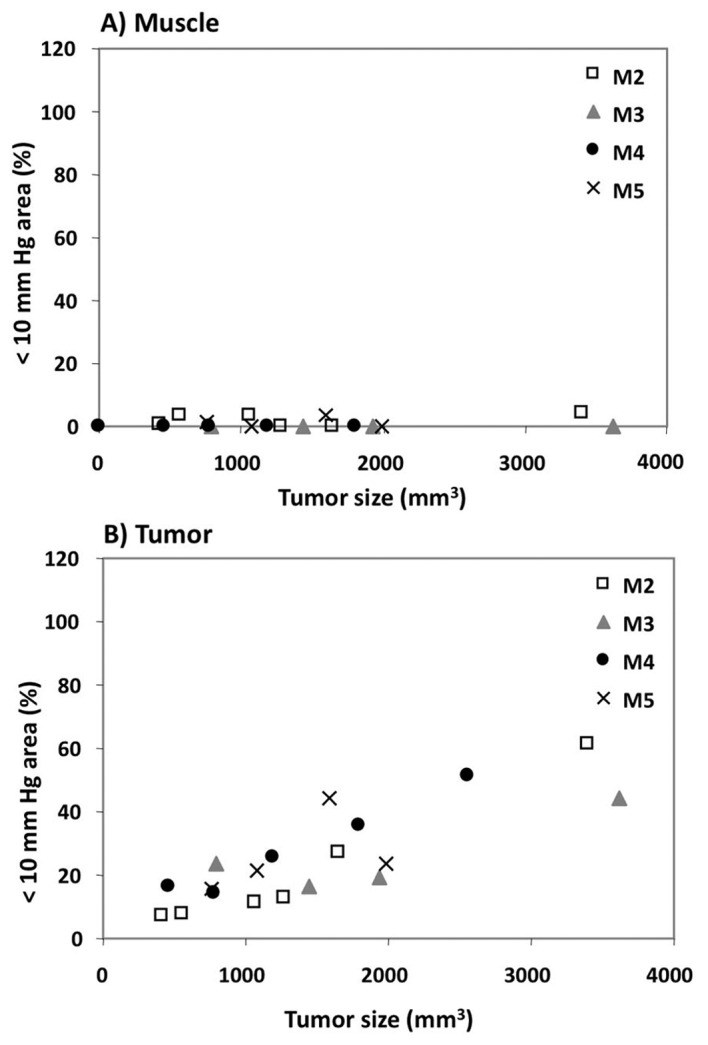
Hypoxic fraction (% of tissue <10 mmHg oxygen) for muscle and SCCVII tumors as a function of tumor size. Normal muscle exhibited little to no hypoxia; whereas, as the SCCVII tumors grew, there was a substantial development of hypoxia. Data presented are for 4 mice followed serially with time.

Between days 7 and 15, the volume of the tumor increased linearly with time from 500 to 3,500 mm^3^. Fig. 1B shows representative EPR-based pO_2_ maps (bottom panels) obtained at 8 and 12 days after tumor implantation. Comparison of the MRI T2-weighted images (top panels) at day 8 and day 12 clearly shows increase in tumor size with time. The pO_2_ maps overlaid with the MRI scans show significant hypoxia regions (black and blue areas on the images) within the tumor by day 12. The pO_2_ maps as shown in Fig. 1B were converted into histograms and representative histograms for a representative mouse on days 7 and 15 are displayed in Fig. 2. These histograms show that over the course of the study, a slight 3 mmHg decrease in median pO_2_ was observed in the muscle, while a marked 8 mmHg decrease in median pO_2_ was observed in the tumor. To test if the hypoxic fraction of the tumor changed as the tumor grew, the percentage of tumor volume with pO_2_ less than 10 mmHg (i.e., the hypoxic fraction) was plotted as a function of tumor size (Fig. 3). Between days 7 and 15 of the study, the hypoxic fraction of the muscle remained well below 5%. In contrast, the hypoxic fraction of the tumor increased linearly as the tumor progressed, reaching approximately 50% by the end of the study. Taken together with the data from Figs. 1 and 2, these results strongly suggest that growth of the SCCVII tumor correlates with the development of hypoxic regions within the tumor.

### The redox status of the tissue was monitored using Tempol as a redox-sensitive magnetic resonance imaging contrast agent

**Figure 4:**
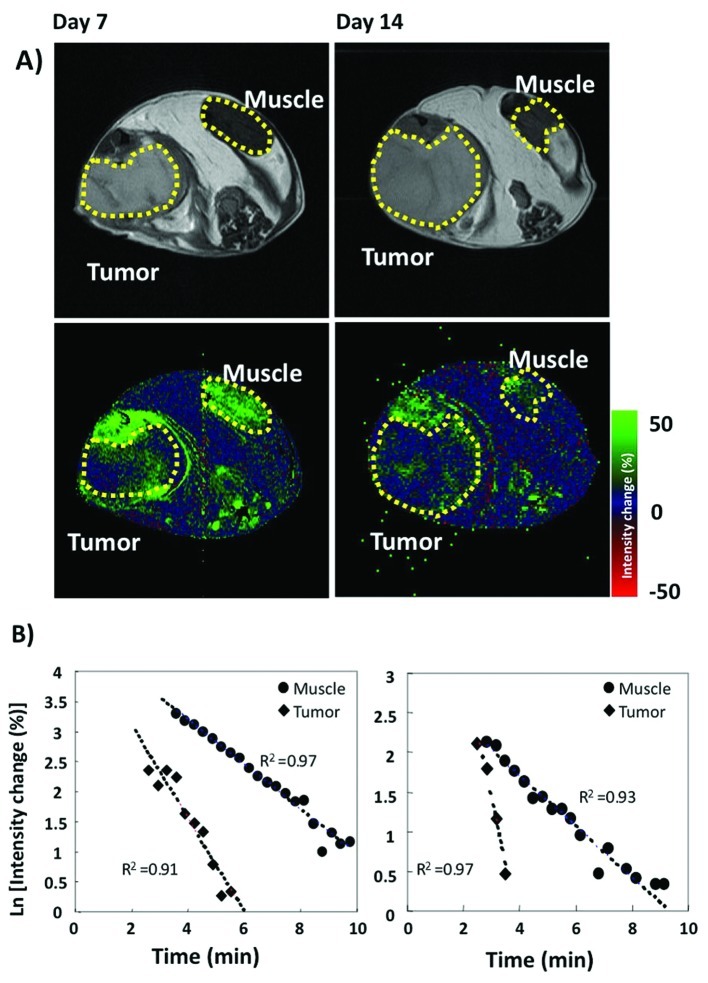
(A) T2-weighted anatomic images of a representative SCCVII tumor-bearing mouse at day 7 and day 14 (upper panels) and corresponding Tempol redox maps (bottom panels) acquired by MRI. Tumor and muscle locations are delineated by dashed yellow line on each of the images. (B) The rate of Tempol decay as a function of time after injection for muscle and tumor ROIs. Linear regression lines are shown on the plots with the respective R2 values indicated.

**Figure 5:**
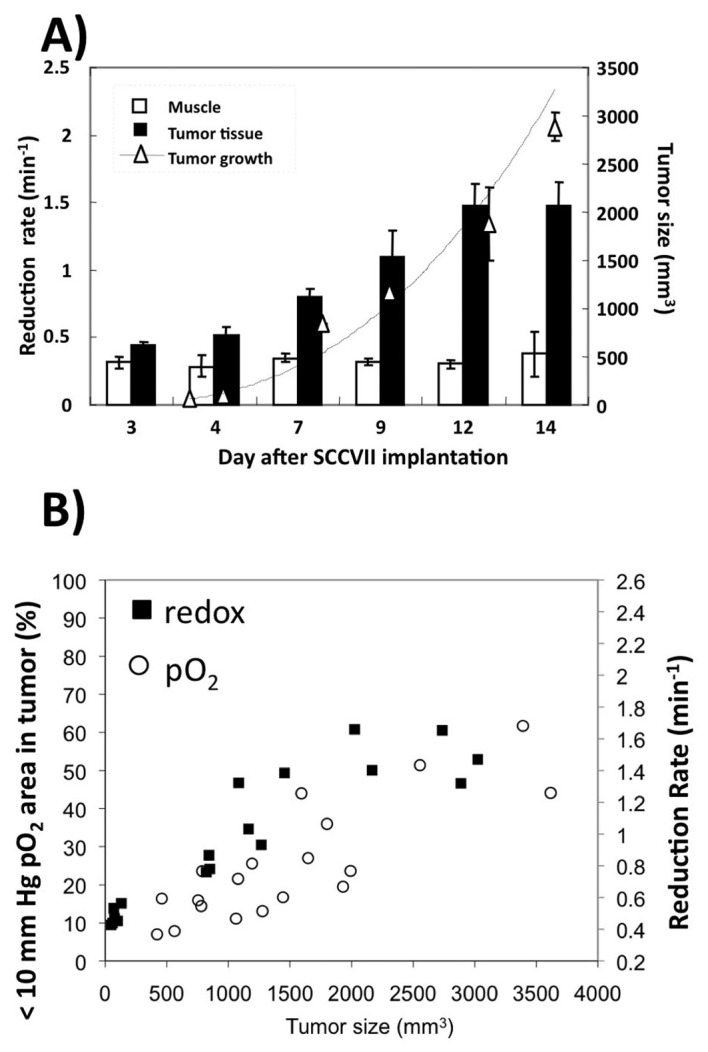
(A) Relationship between Tempol reduction rate and tumor size for muscle and tumor tissue as a function of time, n=4. (B) Relationship between hypoxia (% of tumor <10 mmHg) and Tempol reduction rates as a function of tumor size.

Fig. 4 shows a representative redox data set for a mouse taken at days 7 and 14 after tumor implantation. Fig. 4A shows T2-weighted images of the tumor region, with regions of interest (ROI) outlined with yellow lines for muscle and tumor. ROIs were used to calculate the rate of Tempol reduction. Fig. 4B shows the observed signal enhancement 40 sec after Tempol injection. On both days 7 and 14, the rate of Tempol reduction was greater in tumor tissue than in muscle. As the tumor grew in size, the rate at which muscle reduced Tempol did not appreciably change, while the rate at which the tumor reduced Tempol more than doubled. Fig. 5A summarizes the changes in tumor volume and Tempol reduction rates for 4 mice. As the tumor volume increased, the rate at which the tumor reduced Tempol also increased. As for muscle, Tempol reduction essentially the same over the 14 days. Finally, the rate of Tempol reduction and the hypoxic fraction of a SCCVII tumor were plotted as a function of tumor size as shown on Fig. 5B. This graph shows that larger tumors have hypoxic fractions ranging from 40%–60% and reduction rates varying from 1.2–1.6 min^−1^, while smaller tumors have hypoxic fractions of only 5–20% and reduction rates of only 0.4–0.6 min^−1^. This comparison suggests that the rate of Tempol reduction and the hypoxic fraction of a tumor were positively correlated.

## Discussion

The purpose of this study was to determine if the hypoxic fraction of a SCCVII tumor correlated with the rate of Tempol reduction in the tumor. It was found that both the rate of Tempol reduction as well as the hypoxic fraction (the fraction of voxels with less than 10 mmHg) increased as the tumor grew (Fig. 5B). This implies a correlation between the rate of Tempol reduction and the hypoxic fraction. Because high tumor hypoxia predicts a decreased likelihood of survival during clinical cancer therapy (1–3), the data from the current study suggest that rapid Tempol reduction may be indicative of a poor prognosis for cancer therapy.

In this study, it was noted that the tumor hypoxic fraction generally increased as the tumor size increased, in agreement with other published studies (11,12). Early studies showing a relationship between tumor size and hypoxic fraction used a radiobiological assay of hypoxia (11). One of the most common radiobiological assays of hypoxia involves performing an *in vivo*/*in vitro* colony-forming assay on two experimental groups: mice breathing ambient air, and mice asphyxiated with nitrogen gas. The hypoxic fraction is calculated from the difference in cell survival between the air breathing group and the asphyxiated hypoxic group. Using assays such as these, it was generally found that in a variety of tumors weighing less than a gram, larger tumors exhibited more hypoxia than smaller tumors (11,13–15). In the case of KHT sarcomas, it was noted that tumors larger than 0.7 g did not show a further increase in hypoxic fraction, indicating that some tumors may reach a plateau in their hypoxic fraction (14). In contrast with these studies, studies with both a rhabdosarcoma (16) and in a 9L line (17) were not able to show a dependence of hypoxic fraction on tumor size. Later studies used an invasive polargraphic needle electrode to assess the dependence of hypoxic fraction on tumor size. These studies found that in OCa-I, MCa-r, KHT, C3H mammary carcinoma and SCCVII tumors with weights ranging from 0.15 to 1.5 g, the hypoxic fraction increased as the tumor grew (18–20). In the case of SCCVII (also used in this study), polarographic oxygen measurements showed that the hypoxic fraction (defined in that study as % volume with pO_2_ <5 mmHg) ranged from approximately 50 to 100% as the tumor grew from 150 mm^3^ to 1,500 mm^3^(20). These measurements report hypoxia fractions greater than observed in the present study, where the hypoxic fraction (defined in this study as % volume with pO_2_ <10 mmHg) was found to increase from 10 to 50% as the tumor grew from 500 mm^3^ to 3,500 mm^3^. In summary, most of the literature is consistent with the current finding that implanted animal tumors weighing less than a few grams exhibit an increased hypoxic fraction as the tumors grow.

Although most murine tumors exhibit a dependence of hypoxic fraction on tumor size, there are data suggesting that in the case of human tumors, the situation is more complex. Using F-MISO PET, hypoxia was measured in tumors of 12 patients with non-small cell lung cancer, where the tumor size ranged from 50 cm^3^ to 350 cm^3^(21). In this study, no dependence of hypoxic fraction on tumor size was observed. In other studies in human uterine cervix cancers (22) and squamous cell carcinoma metastasis (23), hypoxia was measured using a polarographic needle. Again, no dependence of hypoxic fraction on tumor size was observed in either of these studies. Although these data are limited, they suggest that tumor size is not a valid single surrogate marker for hypoxia in the clinical setting. It is not completely clear why animal tumors exhibit a size-dependent hypoxia relationship and human tumors do not, but the difference may be related to the relative volume of murine versus human tumors. In general, the human tumors were at least 5 g (assuming water density) (11,13,14), indicating that by the time human tumors are observed in the clinic, they may have surpassed a threshold for size-hypoxia dependence. Indeed, the hypoxic fraction cannot increase indefinitely, and murine KHT sarcomas exhibited a plateau in hypoxia above a weight of 0.7 g (14). Because tumor size, which is commonly measured during routine clinical scans, is apparently a poor marker for tumor hypoxia, novel biological imaging techniques must be developed in order to assess tumor hypoxia in humans.

During this study, it was observed that tumor hypoxia and the rate of Tempol reduction both increased as the tumor grew. The question remains if the increasing hypoxia observed during tumor growth actually caused Tempol to be reduced more quickly. In theory, it is possible that low oxygen levels in a tumor will result in a more reductive tumor microenvironment, and an important example involves the NADPH/NADP^+^ redox pair (24). NADPH is an endogenous antioxidant, and its oxidation is catalyzed by an enzyme called NADPH oxidase (NOX) in a reaction that requires molecular oxygen. Furthermore, NADPH oxidase is overexpressed in some cancers (25), including SCC (26) (used in this study), implying that NOX activity may have affected NADPH levels in the tumor models used during this study. NADPH, in turn, is coupled to the reduction of GSSG to glutathione, which is the most abundant endogenous anti-oxidant. Thus, oxygen levels directly and indirectly may affect the intracellular levels of NADPH and GSH, both of which in turn affect the rate of Tempol reduction (7,27–30). Hypoxia may therefore cause the cellular milieu to become more reduced, which would result in a high rate constant for Tempol reduction.

Although increasing hypoxia may cause the intracellular environment to become more reduced through the mechanisms described above, those effects alone cannot account for the large increase in tumor redox observed in the current study. To put in perspective the findings of this study, it is useful to cite *in vitro* studies showing that compared to tumor cells exposed to 160 mmHg oxygen, cells exposed to 0 mmHg oxygen exhibit a 20–160% greater reduction rate constant (31,32). Furthermore, in a RIF-1 tumor, it was found that the tumor pO_2_ varied between 2.5–15 mmHg, but that the correlation between the nitroxide reduction rate constant and pO_2_ was weak (r=0.357) (33). It therefore seems unlikely that a small change in median pO_2_ of 5 mmHg would result in a 100% increase in the reduction rate constant due only to the mechanisms described in the previous paragraph. These considerations suggest that the rapid Tempol reduction observed during this study may be due to factors more complex than simple accumulation of oxygen-dependent antioxidants due to low levels of molecular oxygen.

An alternative explanation of the apparent correlation between the hypoxic fraction and the rate of Tempol reduction observed in the current study could be a hypoxia-induced shift to glycolytic metabolism. Under conditions of hypoxia, signaling molecules such as hypoxia inducible factors (HIFs) become relatively abundant in the cellular milieu. The most commonly studied hypoxia inducible factor, HIF-1, is known to activate glycolytic enzymes and genes, thereby increasing glycolytic metabolism (34–36). Recently, glycolytic metabolism has been shown to provide resistance to H_2_O_2_ toxicity, decrease production of reactive oxygen species, and decrease the extent of oxidized DNA fragments. In other words, glycolytic metabolism has been shown to evoke an antioxidant response (34–36). Although the mechanism for this antioxidant response is not yet fully understood, it may also contribute to the more reductive tumor environment that was observed in this study.

In this study, it is observed that both hypoxia and the rate of nitroxide reduction increased as the tumor grew, suggesting that hypoxia and the rate of nitroxide reduction are correlated. Due to the prognostic value of hypoxia, this suggests that rapid nitroxide reduction may indicate poor response to cancer therapy. In conclusion, nitroxides such as Tempol may provide a clinically feasible magnetic resonance imaging based assay of hypoxia.
